# Reorganization of 3D genome architecture provides insights into pathogenesis of early fatty liver disease in laying hens

**DOI:** 10.1186/s40104-024-01001-y

**Published:** 2024-03-07

**Authors:** Yanli Liu, Zhuqing Zheng, Chaohui Wang, Yumeng Wang, Xi Sun, Zhouzheng Ren, Xin Yang, Xiaojun Yang

**Affiliations:** 1 College of Animal Science and Technology, Northwest A&F University, Yangling, 712100 China; 2 Institute of Agricultural Biotechnology, Jingchu University of Technology, Jingmen, 448000 China

**Keywords:** 3D chromatin architecture, Fatty liver disease, Folate, H3K27ac profiling, Transcription reprogramming

## Abstract

**Background:**

Fatty liver disease causes huge economic losses in the poultry industry due to its high occurrence and lethality rate. Three-dimensional (3D) chromatin architecture takes part in disease processing by regulating transcriptional reprogramming. The study is carried out to investigate the alterations of hepatic 3D genome and H3K27ac profiling in early fatty liver (FLS) and reveal their effect on hepatic transcriptional reprogramming in laying hens.

**Results:**

Results show that FLS model is constructed with obvious phenotypes including hepatic visible lipid deposition as well as higher total triglyceride and cholesterol in serum. A/B compartment switching, topologically associating domain (TAD) and chromatin loop changes are identified by high-throughput/resolution chromosome conformation capture (HiC) technology. Targeted genes of these alternations in hepatic 3D genome organization significantly enrich pathways related to lipid metabolism and hepatic damage. H3K27ac differential peaks and differential expression genes (DEGs) identified through RNA-seq analysis are also enriched in these pathways. Notably, certain DEGs are found to correspond with changes in 3D chromatin structure and H3K27ac binding in their promoters. DNA motif analysis reveals that candidate transcription factors are implicated in regulating transcriptional reprogramming. Furthermore, disturbed folate metabolism is observed, as evidenced by lower folate levels and altered enzyme expression.

**Conclusion:**

Our findings establish a link between transcriptional reprogramming changes and 3D chromatin structure variations during early FLS formation, which provides candidate transcription factors and folate as targets for FLS prevention or treatment.

**Supplementary Information:**

The online version contains supplementary material available at 10.1186/s40104-024-01001-y.

## Background

Fatty liver disease (FLS) comprises a wide range of pathogenetic process, spanning from simple steatosis with no inflammation to steatohepatitis and liver fibrosis of varying severity, ultimately progressing to cirrhosis, which is a kind of pandemic metabolic disorder in both humans and laying hens. The prevalence of non-alcoholic fatty liver disease (NAFLD) in human beings varies from 23% to 32% across different geographical regions [1]. While in laying hens, it ranges from 16% to 25% [2]. In addition, the main site of de novo lipogenesis is the liver in both humans and chickens. The chicken has been regarded as a suitable model for studying human NAFLD due to their similar incidence and lipogenesis characteristics [3–5]. On the other hand, the mortality rate of laying hens increases from 0.8% at 32 weeks over 10% at 72 weeks, where 74% of dead caged hens were diagnosed with FLS [6], implying that FLS significantly impacts egg-laying performance and poses an urgent problem for the poultry industry.

It was reported that approximately 10%–20% individual at each stage of FLS disease may progress to the next stage, but there is some reversibility, especially in the early periods, such as simple steatosis [7]. Therefore, it is crucial to understand the pathogenesis of FLS during the early stage and identify appropriate regulatory targets to reduce economic losses as early as possible. FLS models in chickens could be induced by high fat [8], high-energy and low protein [9, 10] or methionine choline-deficient [2] diets. These dietary interventions usually need more than 8 weeks and FLS models are accompanied with inflammation phenomenon. In addition, hormonal disruption has been observed in most FLS models induced by diets. Therefore, hormone treatment was considered as a direct method to establish FLS models [9]. As a kind of glucocorticoids known for its anti-inflammatory properties, the dexamethasone (DXM) has been successfully used to induce FLS models in chickens [11], mice [12] and zebrafish [13] with a relatively short time. Hence, DXM was chosen to construct an early FLS model without inflammation in the current study to explore the pathogenesis in laying hens.

The advent of omics technologies has significantly advanced research on the pathogenesis of FLS. Transcriptomics [12], proteomics [5], metabolomics [3, 14], metagenomic or 16S rRNA sequencing [4, 15] have been employed to identify genes, proteins, metabolites or gut microbiota related to FLS. Various metabolic processes have been implicated in FLS development including oxidative stress, endoplasmic reticulum stress, inflammatory response and cell apoptosis [16]. Targeting any pathways involved in these metabolic processes could partially alleviate FLS symptoms. We come up the hypothesis that whether there exist core targets at the genomic level which might regulate multiple pathways simultaneously. Chromosomal three-dimensional (3D) structure, comprising the switching of A or B compartments, reorganization of topologically associated domains (TADs), and enhancer-promoter loops (E-P loops), plays a crucial role in cell development and disease formation by regulating transcriptional reprogramming [17, 18]. Previous studies have reported dynamic changes in 3D chromatin architecture during chicken folliculogenesis [19] and spermatogonial stem cells differentiation [20] using high-throughput chromosome conformation capture (HiC) technology. HiC has also been applied to reveal the regulatory mechanism in porcine adipose tissues [21] and genetic adaptation to extreme environments in chickens [22]. In the present study, we investigated hepatic 3D genome alterations, H3K27ac modifications profiling, and transcriptome changes in chickens with early FLS to systematically reveal the epigenetic mechanisms, aiming to provide insights into exploring core regulatory targets and nutritional regulation strategies of FLS.

## Methods

### Animals and sample collections

Hy-Line Brown layers with the age of 20 weeks were subjected to either normal saline (Con group) or dexamethasone (DXM group) treatment via subcutaneous neck injection at a dosage of 4.50 mg/kg body weight for consecutive 7 d (at 8:00–08:30 once a day) referring to previous report [11]. There were 7 birds for each group and the diet used in the study was the same. After 7 d treatment, blood samples were gain from wing vein for serum collection, which was used for biochemical parameters analysis via Hitachi-7180 automatic biochemical analyzer at Yangling Demonstration Zone Hospital (China). The body weight of each bird was recorded before being sacrificed. After sacrifice, the liver was carefully removed and weighed to calculate the liver index (g of organ/kg of body weight). A portion of the liver was then rapidly frozen using liquid nitrogen and stored at −80 °C for further analysis. The rest was cut into 1 cm^3^ piece and fixed in 4% formaldehyde for morphology detection using hematoxylin-eosin (HE) and Oil Red O staining, which was performed by Wuhan Servicebio technology Co., Ltd. (Wuhan, China). All animal protocols (DK2022007) were approved by the Animal Care and Use Committee of the College of Animal Science and Technology of the Northwest A&F University (Shaanxi, China).

### Hepatic TG and TC contents

A piece of frozen liver tissue was homogenized in cold PBS buffer. The supernatant after centrifugation was obtained to determine protein concentration using BCA protein quantitation kit (AccuRef Scientific, Xi’an, China). Meanwhile, the supernatant was used for total triglyceride (TG) and cholesterol (TC) detection based on the instruction from commercial kits (Nanjing Jiancheng Bioengineering Institute, Nanjing, China), and the results were normalized by protein concentration and showed as per g protein.

### HiC library construction and data processing

Two independent liver samples from each group were used for in situ HiC library construction based on the previously published protocol with several modifications [21, 22], which was performed by Wuhan Yingzi Gene Technology Co., Ltd. (China). Briefly, liver samples were cross-linked for 30 min at room temperature with 1% formaldehyde and the crosslink was quenched in final 0.25 mol/L glycine for 5 min at room temperature. Then fixed livers were homogenized in lysis buffer supplemented with protease inhibitor and incubated at 4 °C for 30 min with rotation. Cell nuclei were collected for permeabilization treatment using sodium dodecyl sulfate and Triton X-100. Furtherly, chromatin was digested with MboI restriction enzyme at 37 °C for 2 h with rotation, followed by adding biotin labels and ligation reaction. Finally, ligated DNA was purified and sonicated into 300–500 bp, after which biotin labeled DNA fragments were captured for library construction and sequencing on the Illumina NovaSeq 6000 platform with 150 bp paired-end sequencing read lengths.

Clean reads were used to map the gallus reference genome (GRCg7b) to obtain valid pairs interaction using HiC-Pro [23]. The Juicer pipeline was employed to identify information about the *cis*/*trans* interaction ratio and interaction distance; then the Interaction matrix was generated and normalized for genomic compartment A/B, TAD and chromatin loop analysis according to previous reports [18–20]. The identification of A and B compartments at 40 kb resolution was carried out using both principal component analysis (PCA) and gene density information with positive or negative PC1 value as A or B compartment respectively. Subsequently, AB index at the 20 kb bin was used to analyze A/B compartment switching and potential genes expression based on the location of their transcriptional start site (TSS). Insulation score value (IS) was used to identify TAD boundaries and distinguish stable, merge, split, rearrangement and specific TADs [24], and then their boundaries were computed to annotate genes with TSS in 10 kb window upstream and downstream of TAD boundary center. Chromatin loops was analyzed using FitHiC (V2.0.7) software at default parameters: the distance of loop anchor < 2 Mb, *Q*-value < 0.01 and loop contact count > 30; and differential loops (|log_2_FC|≥ 1 and *P* < 0.05) were used for identifying associated genes by interacting promoters.

### RNA-seq and RT-PCR

Liver samples were used to obtain total RNA firstly. After checking RNA quality, they were consigned to Shanghai Personal Biotechnology Co., Ltd. for constructing RNA-seq libraries and sequencing using Illumina NovaSeq. In addition, extracted RNA was reverse transcribed into cDNA for verifying genes expression by RT-PCR method using SYBR Green Pro Taq™ II kit (Agbio, Hunan, China) on the FQD-96A (Bioer, Hangzhou, China). Primers were shown in Table S1. Detailed data processes such as differential expression genes identification, GO and KEGG pathways analysis and calculation of gene expression all referred to our previous report [25, 26].

### H3K27ac labeled CUT-tag analysis

Chromatin H3K27ac epigenomic profiling was performed by H3K27ac (abcam-ab4729, Waltham, USA) labeled CUT-tag assay by Wuhan Frasergen Bioinformatics Co., Ltd. Detailed processed was based on the publication [27] and our previous report [25]. In brief, nuclei were purified from liver samples, then concanavalin A-coated magnetic beads were added. Bead bound nuclei were mixed with digitonin buffer and incubated with H3K27ac antibody. Then the second antibody was used for further incubation to improve protein A binding sites. Next, protein G-Tn5 transposome were added and it could bind to antibody, which guided Tn5 transposome cleave genome around target protein. Target protein and their bound DNA were collected for DNA extraction, which was finally used for libraries construction and sequencing. Clean reads were firstly obtained after quality control to align to the reference chicken genome GRCg7b. Differential peaks and their annotations was based on our previous study [25]. DNA motif analysis of differential peaks was performed by MEME Suite tool using JASPAR database.

### Statistical analysis

All experimental data were expressed as mean ± standard error of the mean (SEM) and analyzed by Student’s *t*-test using the GLM program of SPSS 20.0 (SPSS Inc., Chicago, IL, USA). A probability value less than 0.05 was considered statistically significant. Special methods for omics analysis were mentioned and defined at the corresponding position when introducing methods above.

## Results

### Phenotype identification for FLS model in laying hens

**Fig. 1:**
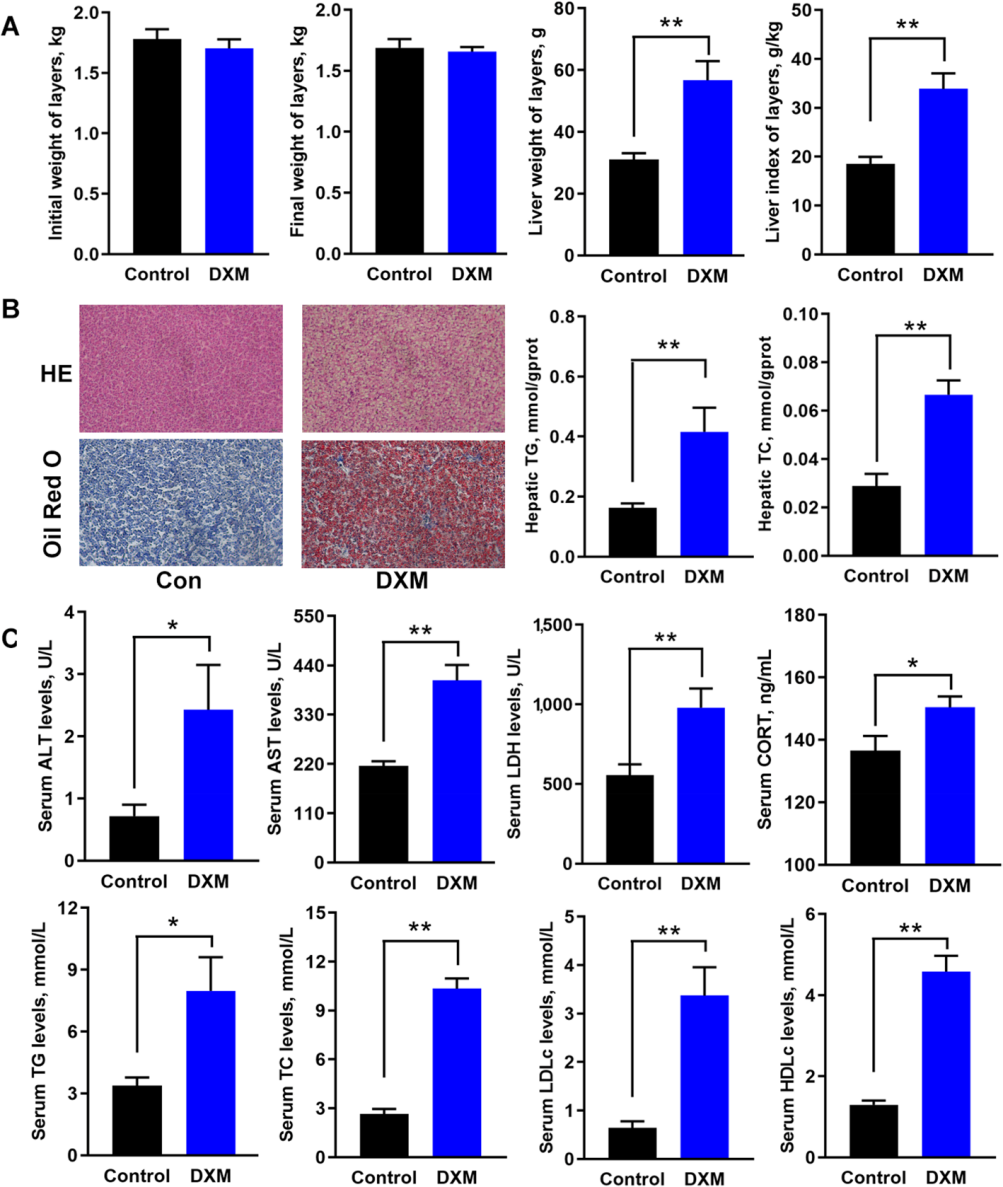
Phenotype observation of laying hens with FLS. **A** Body weight, liver weight and liver index of hens. **B** Hepatic morphological analysis as well as TG and TC detection (magnification:10 × 20). **C** Determinations of serum biochemical parameters. All data are expressed as mean ± SEM (*n* = 7). The asterisks on the bars are statistically significant (* means *P* < 0.05 and ** means *P* < 0.01)

The FLS model was constructed in laying hens by DXM injection. Results showed that DXM injection didn’t affect body weight, while liver weight and index were higher when compared with the Con (Fig. 1A). HE and Oil Red O staining displayed that there were white cavity and red lipid droplets obviously in the liver from FLS group; hepatic TG and TC contents were found to be increased than the Con (Fig. 1B). On the other hand, the analysis of serum biochemical indices indicated that DXM injection increase glutamic-pyruvic transaminase (ALT), aspartate aminotransferase (AST), lactic dehydrogenase (LDH), corticosterone (CORT), TG, TC, low-density lipoprotein cholesterol (LDL-c) and high-density lipoprotein cholesterol (HDL-c) levels (Fig. 1C). These histopathological changes and lipid contents in the liver and serum implied that FLS model was induced successfully.

### Dynamic changes of hepatic 3D chromatin architecture in hens with FLS

**Fig. 2:**
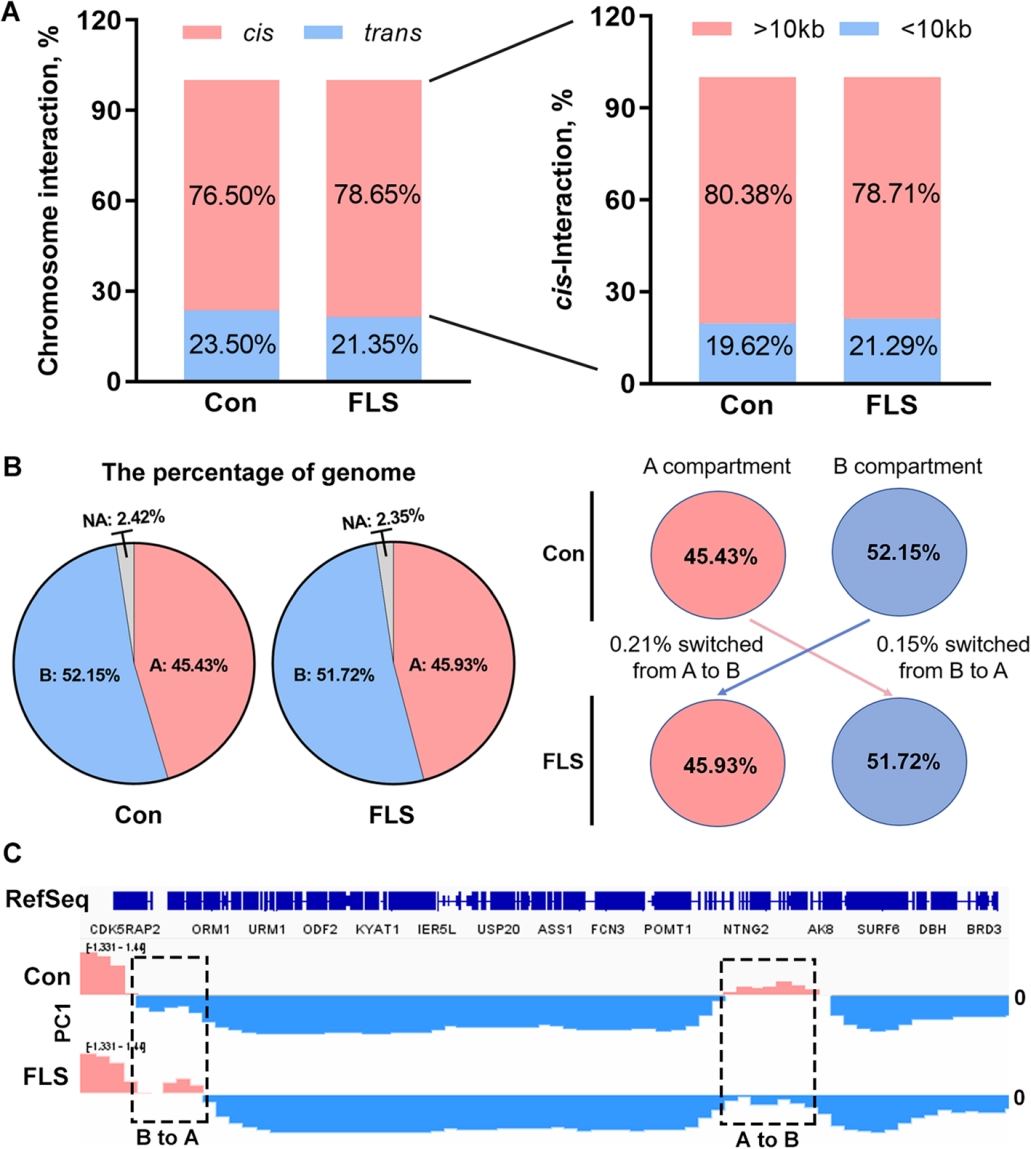
The overview of chromosome interactions and A/B compartment switches in the liver of hens with FLS by HiC. **A** The proportions of interactions between chromosomes (*trans*) or within chromosomes (*cis*). **B** The proportions of A/B compartments and their switching in hepatic genome. **C** The PC1 scores of part genomic regions in Con and FLS groups. The pink parts represent A compartment where the value of PC1 is > 0; and the blue regions mean B compartment where the value of PC1 is < 0

**Fig. 3:**
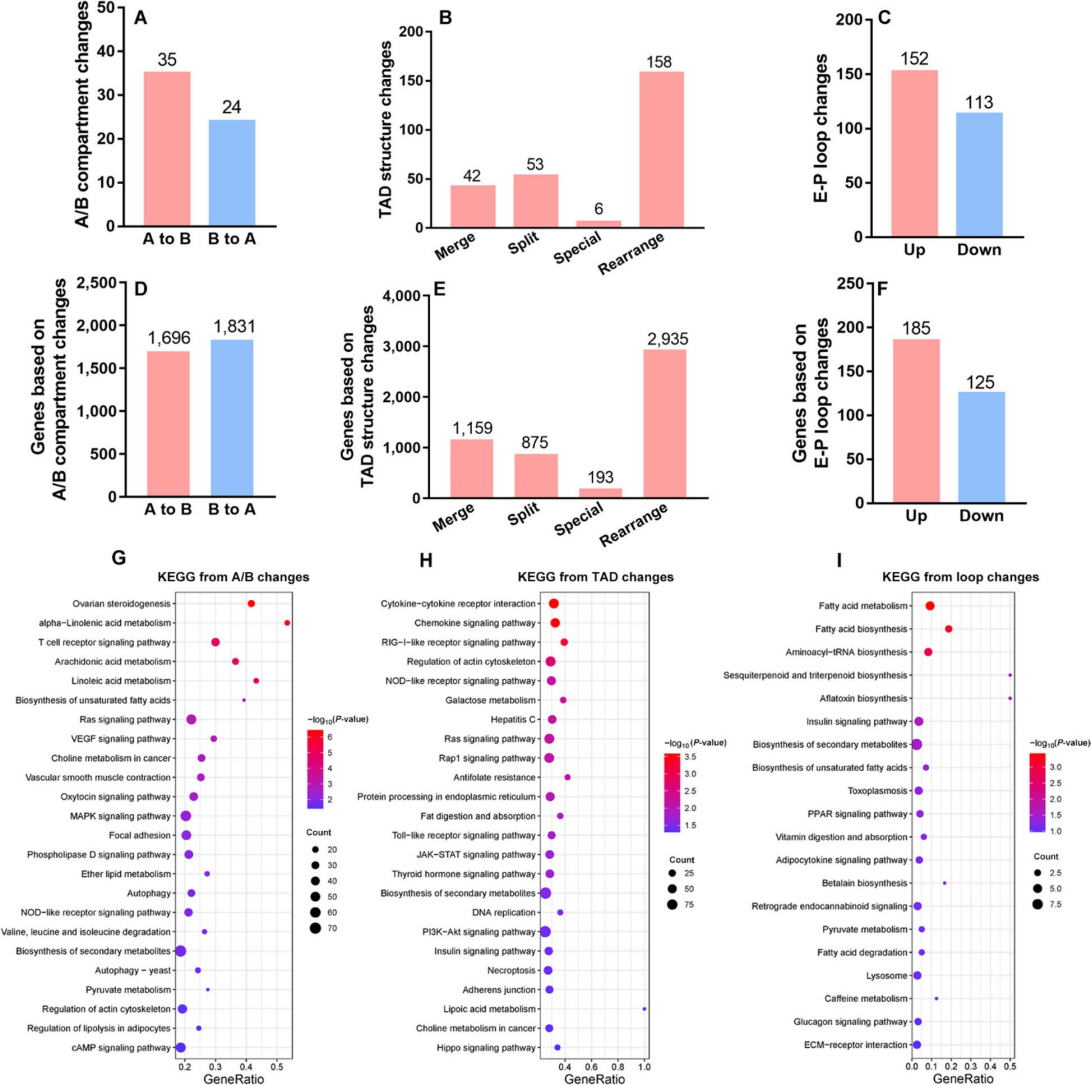
The analysis for hepatic variances in A/B compartments, TADs and chromatin loops as well as their target genes in laying hens with FLS. **A–C** The number of changed A/B compartments, TADs and chromatin loops respectively. **D–F** The number of targeted genes for varied 3D chromatin structure. **G–I** KEGG pathways analysis enriched significantly based on targeted genes from varied 3D chromatin structure

Valid contacts were used to reflect genome interactions. *cis*-Interaction and *trans*-interaction respectively represent intra and inter-chromosomal interaction. As shown in Fig. 2A, the *cis*-interaction occupied most contacts (~ 76.50% in Con and ~ 78.65% in FLS group), among which the dominant (~ 4/5) belongs to long-range interaction (≥ 10 kb). There was no obvious difference in the ratio of *cis*- and *trans*-interaction between Con and FLS group. Then we identified substantial switching levels between actively transcribed compartments A and inactive compartments B based on PCA analysis. 45.43% A and 52.15% B were detected in the Con, whereas 45.93% A and 51.72% B were identified in FLS group; only 0.21% and 0.15% genome were found to undergo A to B and B to A switching respectively when FLS was induced (Fig. 2B). As displayed in Fig. 2C, A/B switching location means genome region labeled with dashed rectangle box where the PC1 value has changed positively or negatively between Con and FLS group. A total of 35 A to B and 24 B to A genome regions were found (Fig. 3A). Similarly, we also analyzed the TAD architecture and chromatin loops changes at a finer scale. As shown in Fig. 3B and C, 259 changed TAD boundaries were found including 42 merges, 53 split, 6 special and 158 rearrangement types in hens with FLS. A total of 265 differential E-P loops also be identified with 152 up and 113 down regulations.

Further, we mapped the genes with genome regions involved in 3D chromatin architecture changes including A/B compartment switching, TAD and E-P loops. Results in Fig. 3D showed that 1,696 genes changed from A to B compartment and 1,831 from B to A compartment during FLS development. Moreover, 5,162 and 310 genes were considered to be associated with changed TAD and differential loops respectively (Fig. 3E and F). Pathway analysis revealed that genes from A/B compartment switching were enriched in fatty acids metabolism, focal adhesion, choline metabolism, Ras, VEGF, and MAPK signaling pathways (Fig. 3G). 5,162 genes involved in TAD changes fell in terms such as Ras signaling pathway, Antifolate resistance, JAK-STAT signaling pathway, adherent junction, DNA replication, insulin and PI3K-AKT signaling pathway (Fig. 3H). Likewise, some fatty acid metabolism, insulin signaling and vitamin metabolism pathways were enriched based on genes from differential loop (Fig. 3I). The detailed information and list of these mapped genes was exhibited in the Additional file 1.

### Genomic H3K27ac profiling and targeted gene analysis

**Fig. 4:**
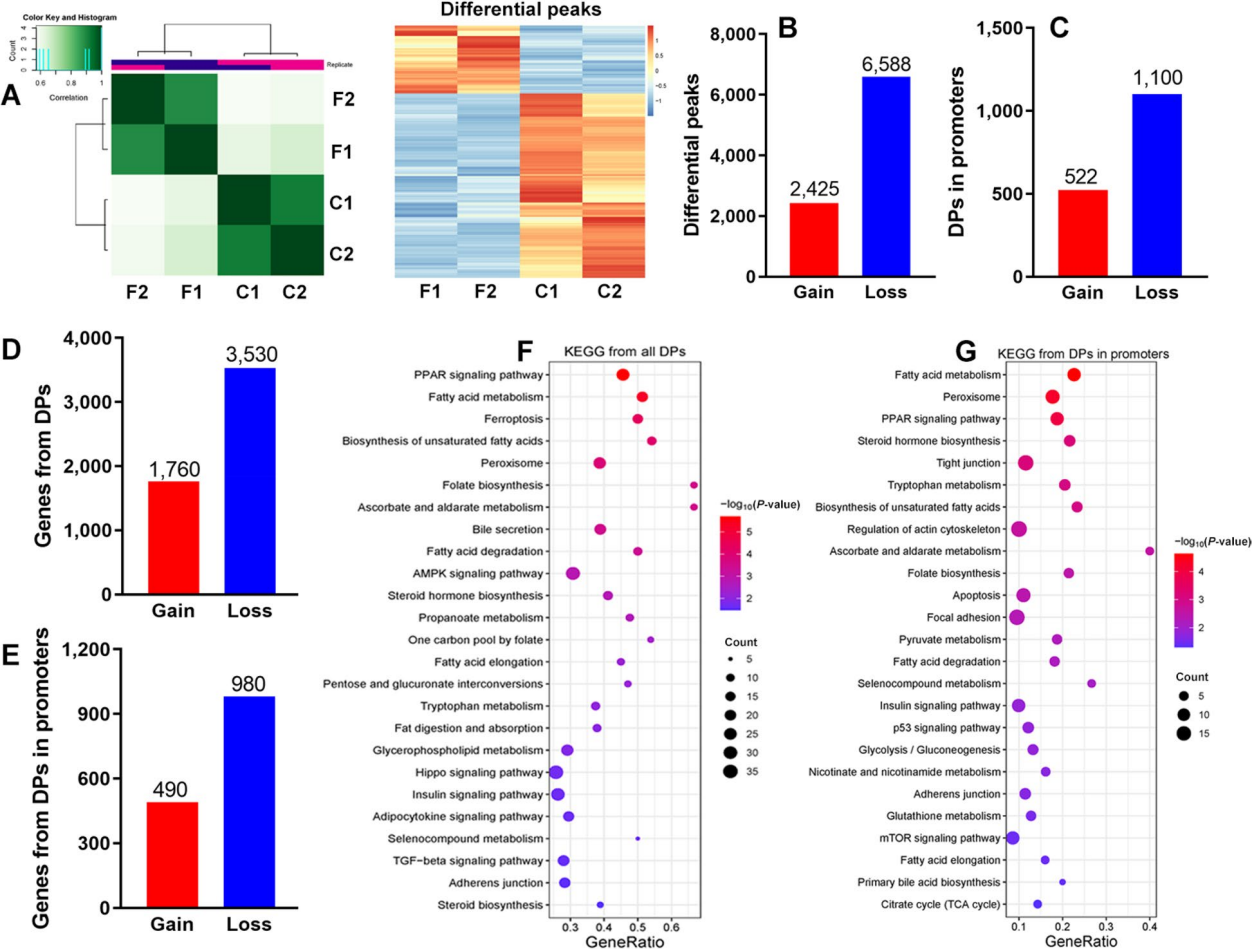
H3K27ac differential peaks and their target genes in Con and FLS groups by CUT-tag analysis. **A** Heatmaps of correlation analysis among samples and H3K27ac differential peaks. **B** and **C** The number of differential peaks in the whole genome and promoters. **D** and **E** The targeted gene numbers of H3K27ac differential peaks. **F** and **G** KEGG pathways analysis enriched significantly based on targeted genes from H3K27ac differential peaks

In order to interrogate the relationship between 3D chromatin architecture and gene transcription, we firstly performed H3K27ac CUT-tag analysis, which is considered as the histone modification for active promoters and enhancers. As exhibited in Fig. 4A, heatmaps for peak correlation and differential peaks among samples showed obvious difference between Con and FLS groups. A total of 2,425 gain and 6,588 loss peaks were identified, among which 522 and 1,100 were located in the promoter region, respectively (Fig. 4B and C). Total gain and loss peaks separately mapped 1,760 and 3,530 genes, while only 1,470 genes were predicted when focusing on differential peaks in the promoter regions (Fig. 4D and E). These mapped genes were listed in the Additional file 1. Whether total mapped genes or gain/loss genes from differential peaks, enrichment analysis found that they were associated with pathways such as fatty acid metabolism, folate metabolism, bile acid metabolism, tight junction or focal adhesion, PPAR signaling, insulin signaling, AMPK signaling, peroxisome, apoptosis and so on (Fig. 4F–G and Fig. S1), which were consistent with those enriched from 3D chromatin architecture changes to some extent.

### Hepatic transcriptional reprogramming changes

**Fig. 5:**
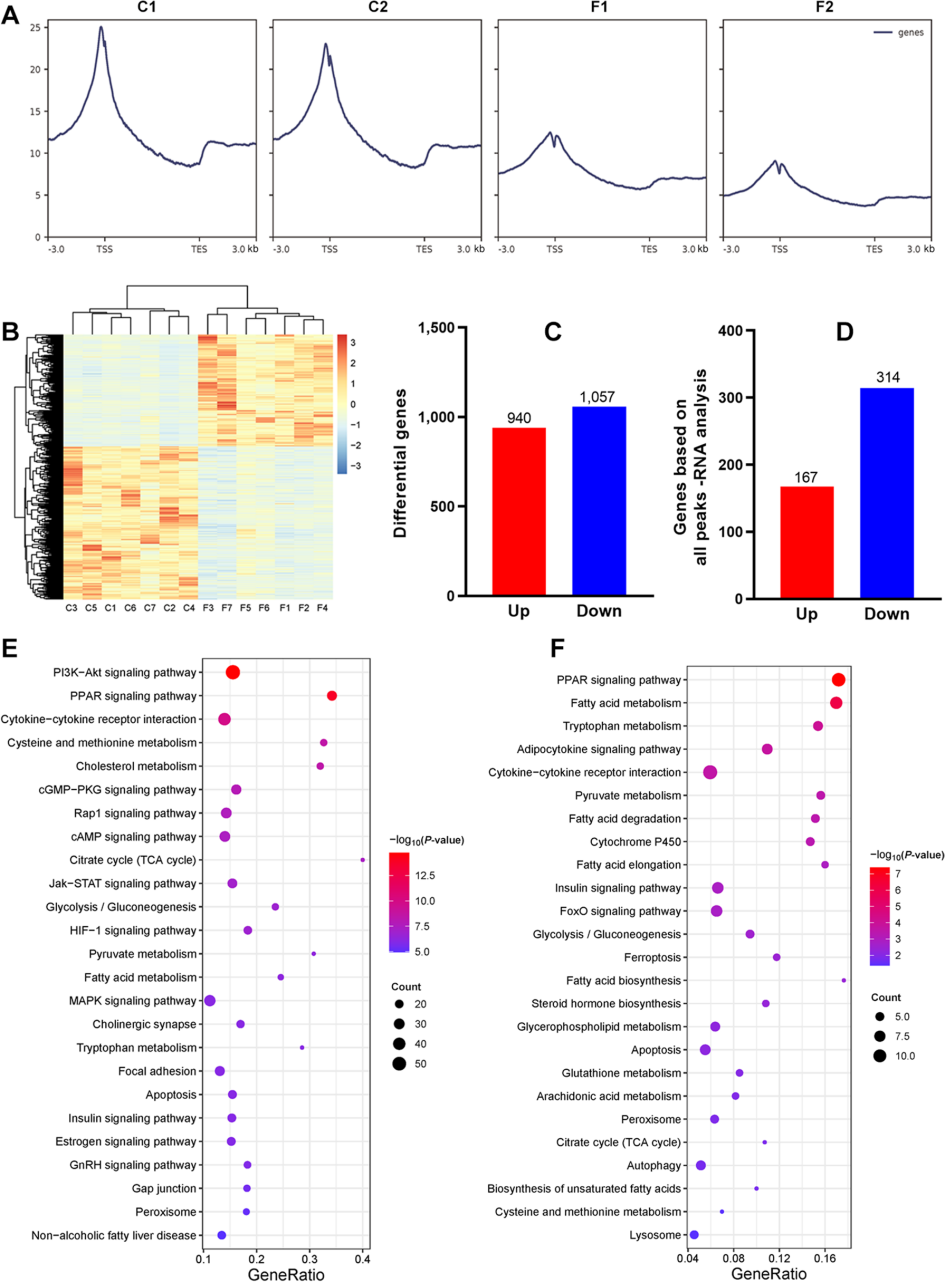
Hepatic transcriptional reprogramming analysis in hens with FLS. **A** H3K27ac signals profiling during 3 kb regions upstream and downstream of the transcription start site in Con and FLS group. **B** and **C** The heatmaps and number of DEGs based on RNA-seq analysis. **D** Overlap analysis of between DEGs and targeted genes from H3K27ac differential peaks in the promoter regions. **E** and **F** KEGG pathways analysis enriched from DEGs and overlapped genes respectively

As shown in Fig. 5A, H3K27ac signals enrichment during 3.0 kb region upstream and downstream of the transcription start site exhibited a relatively large difference between Con and FLS group. To further illustrate transcriptional reprogramming changes when FLS developed in hens, we performed RNA-seq analysis to identify differential expression genes (DEGs). Likewise, the heatmap of gene expression pattern clearly distinguished the two groups (Fig. 5B). 940 up and 1,057 down-regulated genes were found in FLS group when compared with the Con (Fig. 5C). We also overlapped up or down DEGs with predicted genes from corresponding gain or loss H3K27ac peaks (Fig. 5D). 167 overlapped genes between up DEGs and genes from gain peaks genes were found including acetyl-CoA carboxylase alpha (*ACACA*), fatty acid synthase (*FASN*), and elongase of very long chain fatty acids family member 6 (*ELOVL6*), which were related to de novo lipid synthesis; in addition, carnitine palmitoyltransferase 1A (*CPT1A*), microsomal triglyceride transfer protein like (*MTTPL*), poly ADP-ribose polymerase 4 (*PARP4*) and integrin subunit beta 2/6 (*ITGB*2/6) were contained among 314 overlapped genes between down DEGs and genes from loss peaks, which was associated with lipid catabolism, DNA repair or anti-apoptosis and cell integrality. These genes list was showed in Additional file 1. KEGG pathway analysis based on DEGs and overlapped gene were exhibited in Fig. 5E and F respectively; there existed same pathways such as PPAR signaling, fatty acid metabolism, cytokine-cytokine receptor interaction, cysteine and methionine metabolism, citrate cycle, glycolysis/gluconeogenesis, pyruvate metabolism, tryptophan metabolism, apoptosis, insulin signaling and peroxisome, which were similar to those enriched from 3D chromatin architecture.

**Fig. 6:**
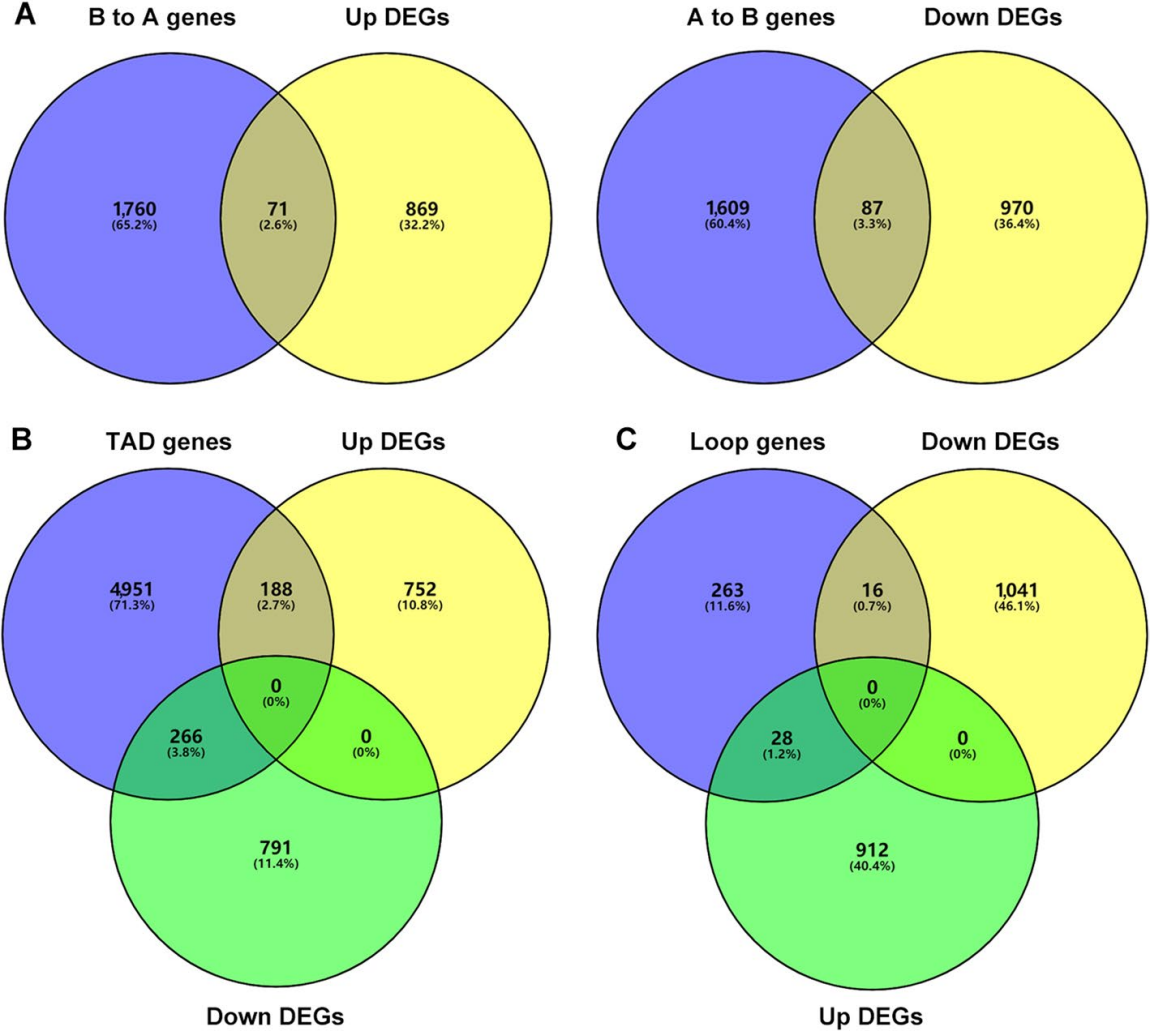
The Venn map of overlap analysis between GEGs and target genes from varied 3D chromatin structure. **A** Genes predicted from changed A/B compartment switching overlapped with the corresponding up or down DEGs. **B** Genes predicted from changed TAD overlapped with up or down DEGs. **C** Genes predicted from differential loops overlapped with up or down DEGs

To further pursue the relationship between transcriptional and 3D chromatin architecture changes, we also performed overlap analysis for DEGs and predicted target genes from 3D chromatin architecture changes. A total of 158 DEGs were confirmed from A/B switching with same trend including 71 B to A and 87 A to B (Fig. 6A). In addition, 188 up DEGs and 266 down DEGs were overlapped with target genes from variational TAD (Fig. 6B). Similarly, there were 16 and 28 genes from differential loops being identified down and up DEGs respectively (Fig. 6C). The detailed information of these overlapped genes was listed in the Additional file 1.

### DNA motifs prediction involved in 3D chromatin remolding

**Fig. 7:**
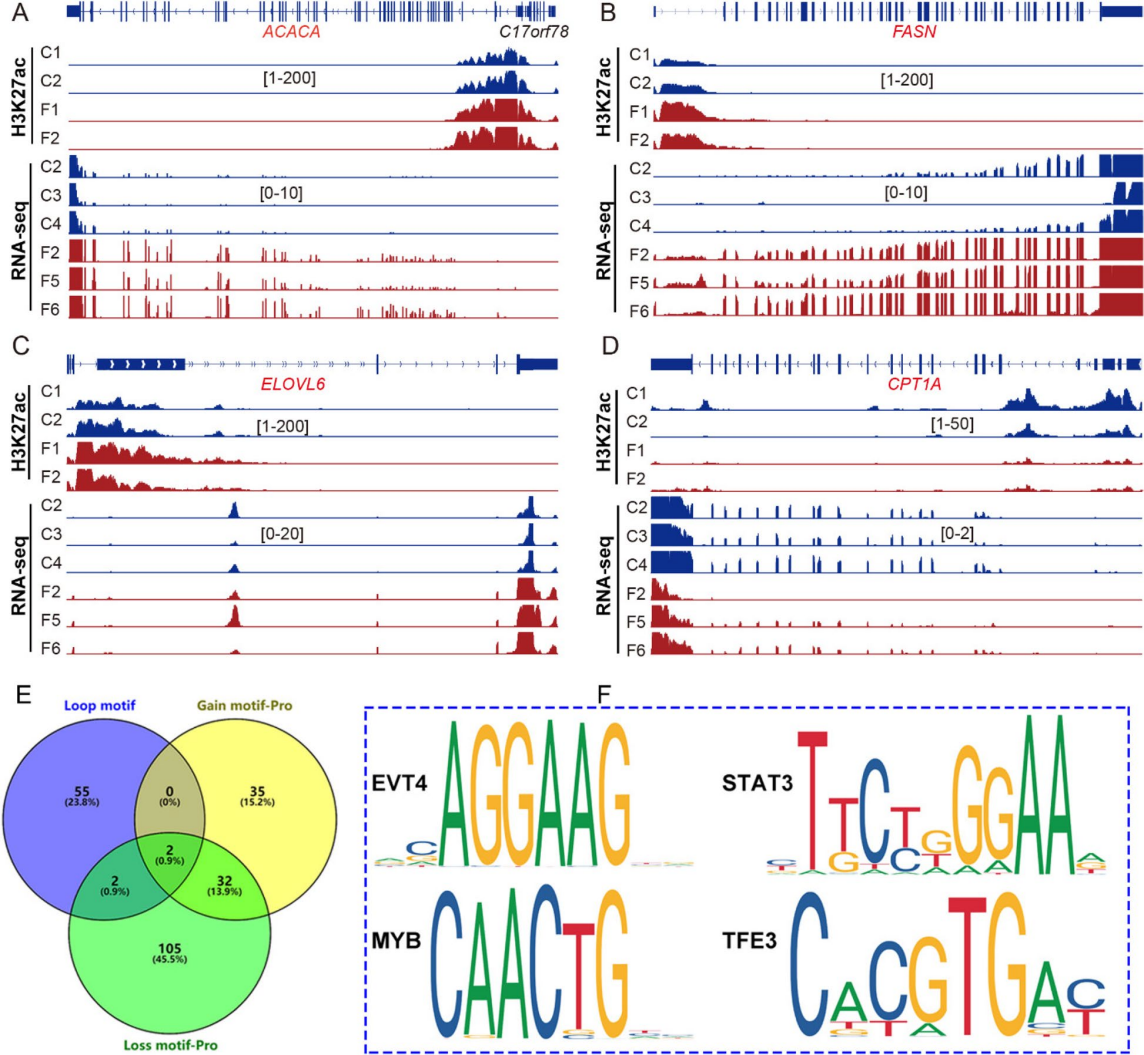
Schematic diagram of H3K27ac differential peaks in the promoters and DNA motifs analysis. **A–D** H3K27ac signal enrichment in the promoter regions and genome tracks of RNA-seq for *ACACA*, *FASN*, *ELOVL6* and *CPT1* genes. **E** The Venn map of DNA motifs predicted from differential gene regions. **F** DNA sequences of overlapped DNA motifs for *EVT4*, *MYB*, *STAT3* and *TFE3*

Transcriptional factors (TFs) are usually considered to take part in 3D genome organization and transcriptional reprogramming by binding DNA and looping TF-bound enhancers to distant target genes. To reveal underlying mechanism on 3D chromatin architecture regulating transcriptional reprogramming, we carried out DNA motif analysis for all differential loops and gain or loss H3K27ac peak regions in gene promoters. Just like Fig. 7A–D, H3K27ac signals enrichment in promoters of *ACACA*, *FASN* and *ELOVL6* genes were significantly higher in FLS group, but the H3K27ac signal was obviously weaker in the promoter of *CPT1A*. RT-PCR results showed that mRNA abundance of *ACACA*, *FASN* and *ELOVL6* were increased while *CPT1A* gene expression was reduced in FLS group (Fig. S2A–D). Based on all differential loops, gain and loss peaks in the promoter regions similar with those above, a total of 59, 69 and 141 TFs were significantly predicted for respectively. The detailed information of predicted TFs was listed in the Additional file 1, among which the top 20 TFs were displayed in Fig. S2E–G. The overlap Venn map for these predicted TFs found that 4 proteins might be involved in loops together with gene promoters including EVT4, STAT3, MYB proto-oncogene MYB, and TFE3 (Fig. 7E–F).

### The nutritional target for FLS intervention from the perspective of genomics

**Fig. 8:**
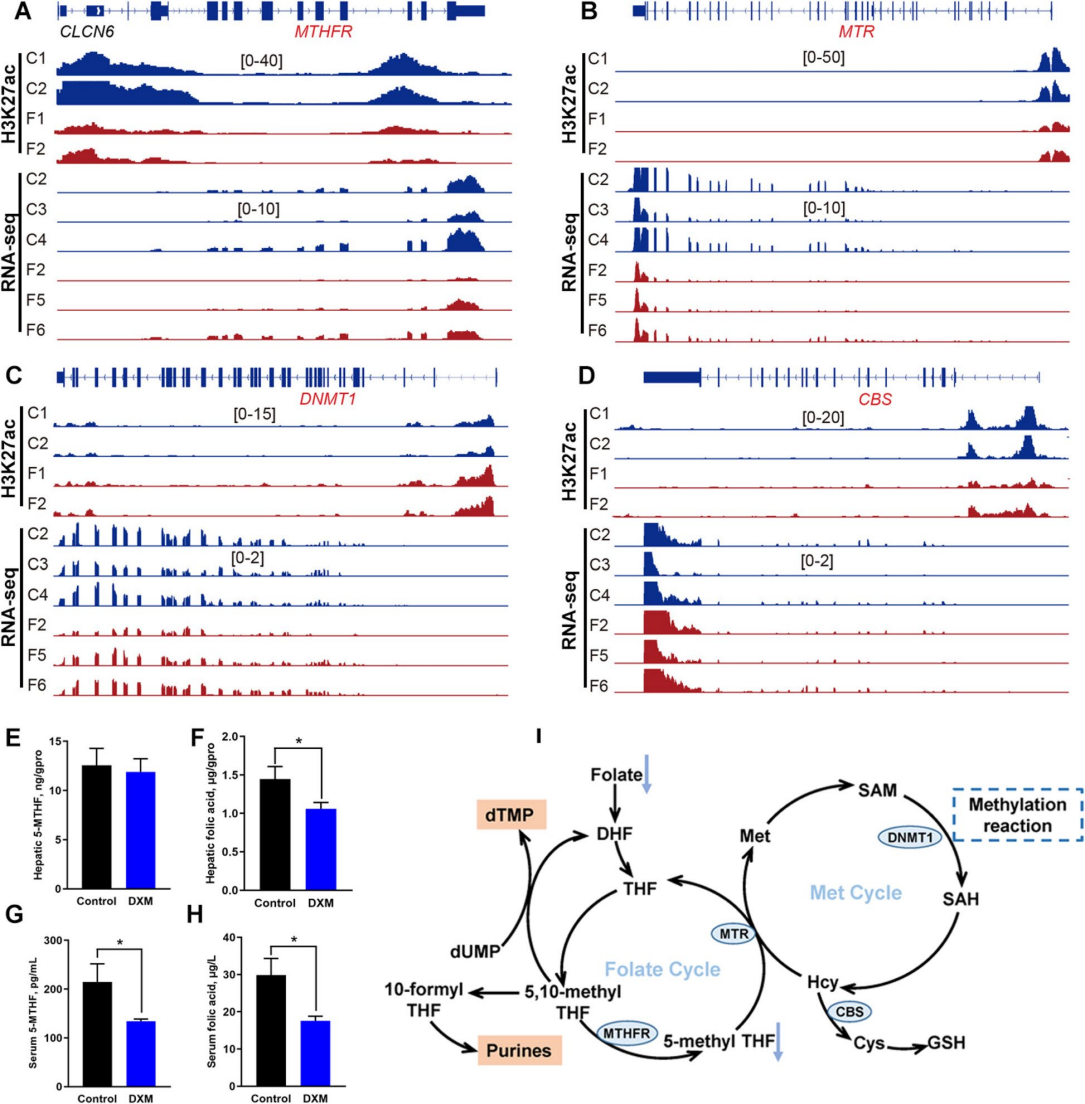
The relationship analysis of folic acid nutritional target for FLS. **A–D** H3K27ac signal enrichment in the promoter regions and genome tracks of RNA-seq for *MTHFR*, *MTRR*, *DNMT1* and *CBS*. **E**–**H** The detection of 5-MTHF and folic acid in the liver and serum. **I** Schematic diagram of folate/methionine cycles and their relationship with FLS. The enzyme expression framed with bule ellipses was down-regulated in hens with FLS

Considering antifolate resistance, folate biosynthesis, and one carbon pool by folate being enriched from changed TAD and differential H3K27ac regions, we focused on metabolic enzymes related to folate/methionine cycles and one carbon metabolism, and found that hepatic H3K27ac signals in the promoter of 5,10-methylene tetrahydrofolate reductase (*MTHFR*), methionine synthetase (*MTR*) and cystathionine-beta-synthase (*CBS*) were weaker in hens with FLS; whereas it was higher in the promoter of DNA methyltransferase 1 (*DNMT1*) (Fig. 8A–D). RT-PCR results showed that *MTHFR*, *MTR*, *DNMT1* and *CBS* genes expression were lower in FLS group when compared with the control (Fig. S2H–K). What’s more, as shown in Fig. 8E–H, folic acid levels in the liver and serum were decreased when FLS occurred in hens. The same phenomenon was found on serum 5-methyl-THF level although no change was detected in the liver.

## Discussion

FLS is a common metabolic syndrome and has been observed to be inheritable in laying hens [2]. Similarly, it was reported that NAFLD in humans was considered a highly heritable trait with estimated heritability ranging from 25% to 60% [16, 28, 29]. Thus, revealing epigenetic mechanism underlying the early formation of FLS not only provides potential targets for nutritional prevention but also holds promise for breeding disease-resistant laying hens. Epigenome technologies such as HiC have been widely applied to elucidate the pathogenesis in various disease, particularly during tumorigenesis. Gene expression regulation is closely linked to 3D chromatin organization in B cell lymphoma [30], epithelial and breast cancer cells [31]. Our previous study also indicated that chromatin looping played a crucial role in shaping transcriptional reprogramming in human epithelial squamous cancers [32, 33]. In this study, we delved into the 3D chromatin organization and its effects on transcriptional reprogramming during the early formation of FLS by integrating Hi-C, RNA-seq and H3K27ac labeled CUT-tag analysis. We induced early FLS in laying hens using neck subcutaneous DXM injection and found increased liver index, visible lipid deposition based on HE and Oil Red O staining, and abnormally elevated TG and TC levels in the liver and serum. However, no obvious inflammation was observed in the liver based on HE morphological analysis. These results implied that hepatic steatosis was induced in laying hens, representing the early stage of FLS.

The occurrence of FLS was accompanied with significant transcriptional reprogramming changes as validated in the present study. RNA-seq analysis revealed that the expression of *ACACA*, *FASN*, *SCD* and *ELOVL6* genes was upregulated in FLS group, which are related to de novo lipogenesis. DXM, being a glucocorticoid, has been reported to regulate the transcription of lipogenic genes through its interaction with the glucocorticoid receptor [11]. Additionally, the expression of *MTTP* and cholesteryl ester transfer protein (*CETP*), responsible for transporting lipids out of the cell, was found to be elevated, indicating a potential compensatory effect due to increased lipid anabolism during the occurrence of FLS. On the other hand, *CPT1A*, which is associated with fatty acid oxidation, was downregulated in hens with FLS. These DEGs related to lipid metabolism corresponded with the observed phenotype of elevated lipids in the liver and serum. Moreover, the findings were consistent with those identified in naturally occurring early FLS hens in a previous study [26], implying that the FLS model used in the study could simulate laying hens with FLS under natural conditions to some extent. Interestingly, no difference was observed in pro-inflammatory cytokines, and *IL-1β* was found to be decreased in hens with FLS. This might be attributed to the anti-inflammatory function of DXM and the relatively short period of FLS induction in the study. However, apart from expected pathways related to lipid metabolism, focal adhesion and gap junction pathways also were enriched from DEGs, suggesting a potential impact of FLS on hepatic integrity. Integrin-α/β superfamily members play important roles in multiple cellular processed including cell proliferation by interacting with the extracellular matrix and the cytoskeleton [34]. Cyclin-dependent kinases (CDKs) were related to the process of cell cycle. *ITGA2B*, *ITGA8*, *ITGB2/6*, *CDK3/6/7* were found to be downregulated. These findings suggested that hepatic integrity was compromised during the occurrence of FLS, even though it was not enough to trigger an inflammatory response, highlighting the importance of protecting liver integrity as a potential target for preventing or alleviating FLS in laying hens.

The reconfirmation of transcriptional reprogramming changes in hens with FLS was achieved through hepatic H3K27ac profiling. H3K27ac is a histone mark associated with active promoters and enhancers, and often used to evaluate gene expression patterns [10, 35]. In the present study, upregulation of *ACACA*, *FASN*, *ELOVL6* and downregulation of *CDK3/6* and *ITGB2/6* were found to be associated with corresponding gain or loss H3K27ac peaks. KEGG analysis based on these differential peaks revealed enrichment in lipid metabolism pathways, such as fatty acid metabolism, biosynthesis of unsaturated fatty acid, PPAR and insulin signaling pathways, which was consistent with the findings from RNA-seq analysis. Likewise, pathways related to hepatic integrity, such as tight junction, focal adhesion and adherent junction, were also observed based on H3K27ac differential peaks in the promoter regions. All of these collectively indicated that hepatic transcriptional reprogramming indeed shifted towards lipid deposition and liver damage during the early occurrence of FLS.

We raise a presumption that there might exist some critical factors which could regulate so many DEGs and lead to hepatic transcriptional reprogramming changes. As we all known, 3D chromatin structure is known to play a role in regulating transcriptional reprogramming during cell development and disease progression [18, 19], we further analyzed dynamic variations in A/B compartments, TADs, and chromatin loops, aiming to gain insight into the contribution of 3D chromatin organization to gene transcription in hens with FLS. Among all DEGs, a total of 158, 454 and 44 DEGs were respectively matched with variations in A/B compartments, TADs and loops, including genes like *FASN*, *ELOVL6* and *MTTP*, implying hepatic transcriptional reprogramming was indeed related to 3D chromatin structure, especially in lipid metabolism. Pathway enrichment analysis based on 3D chromatin structure also illustrated the similarity with those from H3K27ac differential peaks and RNA-seq analysis. Although there might be some differences among the overlapped genes between DEGs and those targeted by 3D genomic structure and H3K27ac binding, it was evident that lipid metabolism and hepatic integrity were disturbed at multiple omics levels.

TFs are essential during the process of 3D chromatin structure regulating gene transcriptions at a long-range distance [32]. DNA motif analysis of various genomic regions in differential loops and H3K27ac enrichment in the promoters were utilized to predict candidate TFs that were involved in transcriptional regulation mediated by chromatin loops during FLS occurrence. Several protein families of TFs, including MYB, SOX, FOXO, KLF, STAT, GATA, HNF and TCF factors, were predicted to be potentially involved in transcriptional regulation through chromatin loops in hens with FLS. TFs overlap analysis between differential loops and H3K27ac gain peaks identified two DNA motifs, *MYB* and *EVT4*. Additionally, *STAT3* and *TFE3* were identified when overlapped with H3K27ac loss peaks. The previous study reported that *STAT3* was intertwined in the reprogramming of lipid metabolism in Hep2 cells under the condition of hypoxia through the JAK/STAT pathway [36]. *MYB* was known to take part in G1/S and G2/M gene expression during the cell cycle in mammals [37], implying it might mainly regulate transcriptional reprogramming of metabolism pathways related to hepatic integrity in hens with FLS. On the other hand, *TFE3*, as a member of the MiT family of the bHLH-leucine zipper TFs, could be involved in the glucose and lipid metabolism via interacting with FOXO1 and recruiting SREBP-1c in the promoter of lipid related genes [38]. The study did not further explore how these predicted TFs shape 3D chromatin structure due to limitations such as the availability of specific antibodies and hepatocyte cell lines in chickens. These predicted TFs could still be considered as potential intervention targets for FLS and candidate genes for breeding chickens with a low incidence of FLS.

An interesting finding regarding folate metabolism provides valuable insights into potential nutritional regulation targets for FLS in laying hens from the perspective of 3D genome organization. The enrichment of folate metabolism based on variations in 3D chromatin structure and H3K27ac peaks, such as antifolate resistance, folate biosynthesis and carbon pool by folate, suggested that folate played a crucial role in the development of FLS. It was reported serum folic acid levels were negatively associated with the presence and severity of liver steatosis in adults [39]. A meta-analysis revealed an inverse association between serum folate and NAFLD risk [40]. Consistent with these findings, our results also confirmed this phenomenon with lower folic acid level in hens with FLS. Moreover, H3K27ac enrichment was lower in the promoter of *MTHFR* and *MTR*, which are important enzymes in folate/methionine metabolism cycles. Except for its role in one carbon metabolism, folate is closely related to purine and thymidylate synthesis as well as amino acid metabolism [41]. It was possible that DNA replication and protein processing was affected due to disturbed folate metabolism in birds with FLS, which further leaded to hepatic damage. The present study firstly reported folate as potential nutritional regulation target from the perspective of 3D genome organization.

## Conclusions

**Fig. 9:**
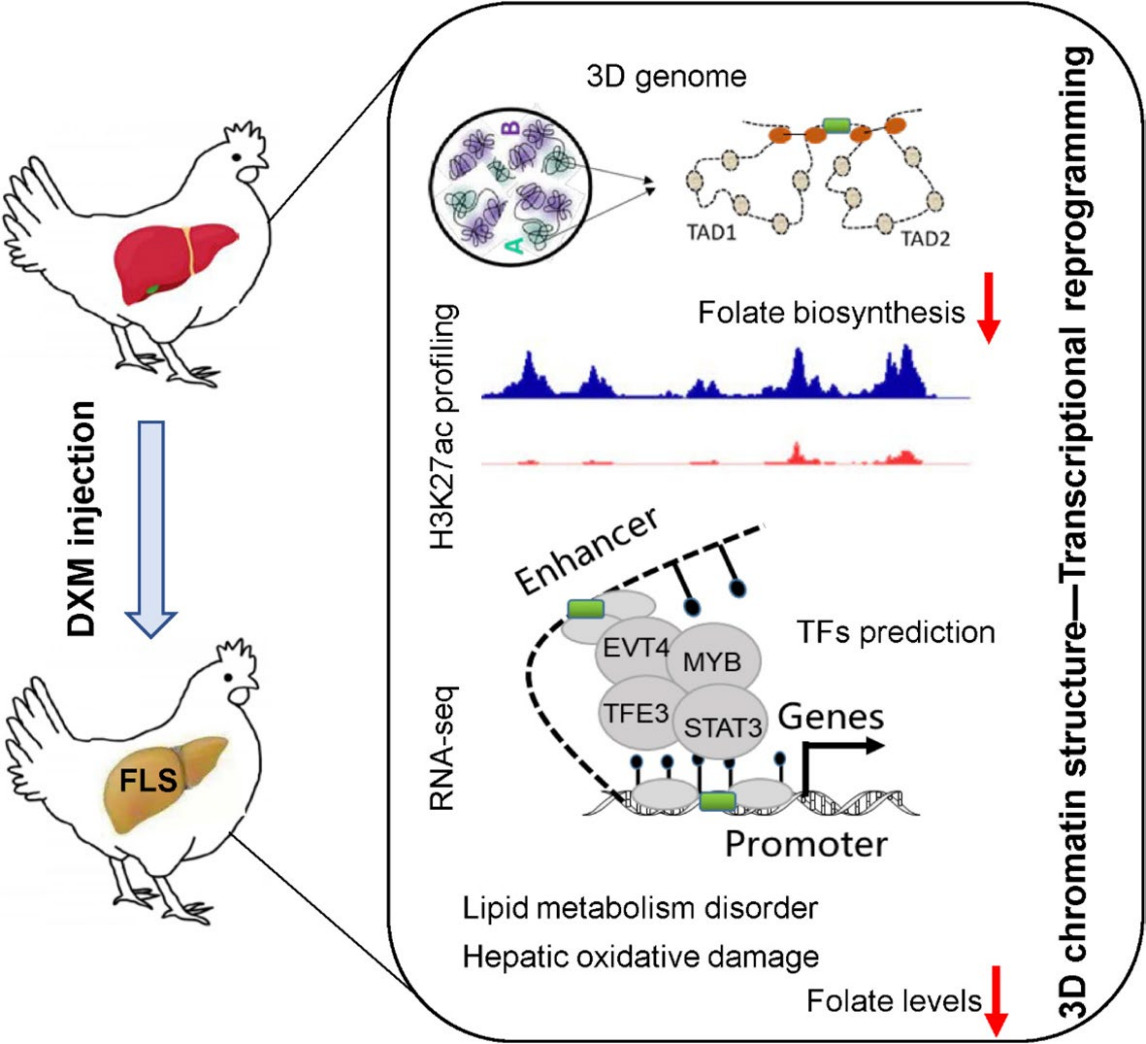
The study constructed early FLS model in laying hens and revealed the potential epigenetic mechanism of early FLS formation by integrating Hi-C, RNA-seq and H3K27ac labeled CUT-tag analysis. Our findings broaden the knowledge of FLS pathogenesis and provided candidate TFs and folate as targets for FLS prevention or treatment

To sum up, the study systematically analyzed alternations in hepatic 3D genome organization and H3K27ac profiling spectrum in laying hens with early FLS, and revealed underlying mechanism about their effects on transcriptional reprogramming. These findings broaden the knowledge of pathogenesis of early FLS and provide candidate TFs and folate as targets for breeding selection and nutritional prevention ways (Fig. 9). Further study is needed to verify how these targets reverse transcriptional changes of FLS by reshaping 3D genome architecture.

### Supplementary Information

**Additional file 1**: Gene lists for 3D genome changes and DEGs as well as their overlap details.

**Additional file 2: Table S1. **Forward and reverse primer sequences for RT-PCR analysis.

**Additional file 3: Fig. S1.** KEGG pathways analysis based targeted genes from gain (**A**) and loss (**B**) H3K27ac peaks as well as differential peaks in promoter regions (**C** for gain and **D** for loss). **Fig. S2.** Gene expression from RT-PCR and DNA motif prediction. **A**–**D** Hepatic *ACACA*, *FASN*, *ELOVL6* and *CPT1A* expression respectively. **E**–**G** DNA motif analysis for differential chromatin loops, loss and gain H3K27ac peaks in the promoter regions respectively. **H**–**K** Hepatic MTHFR, MTR, DNMT1 and CBS expression. All data for gene expression are showed as mean ± SEM (*n *= 7). The asterisks on the bars are statistically significant (* means *P* < 0.05 and ** means *P* < 0.01).

## Data Availability

The author confirmed that all data underlying the findings in the current study are fully available without restriction from the corresponding author on reasonable request.

## Abbreviations

ACACA: Acetyl-CoA carboxylase alpha
ALT: Glutamic-pyruvic transaminase
AST: Aspartate aminotransferase
CBS: Cystathionine-beta-synthase
CORT: Corticosterone
CPT1A: Carnitine palmitoyltransferase 1A
3D: Three-dimensional
DEGs: Differential expression genes
DXM: Dexamethasone
ELOVL6: Elongase of very long chain fatty acids family member 6
E-P: Enhancer-promoter
FASN: Fatty acid synthase
FLS: Fatty liver
HDL-c: High-density lipoprotein cholesterol
HiC: High-throughput/resolution chromosome conformation capture
ITGB: Integrin subunit beta
LDH: Lactic dehydrogenase
LDL-c: Low-density lipoprotein cholesterol
MTHFR: 5,10-Methylene tetrahydrofolate reductase
MTR: Methionine synthetase
MTTPL: Microsomal triglyceride transfer protein like
NAFLD: Non-alcoholic fatty liver disease
PARP4: Poly ADP-ribose polymerase 4
TAD: Topologically associating domain
TC: Total cholesterol
TG: Total triglyceride
TFs: Transcription factors
