# Supplementary material for: Reorganization of 3D genome architecture provides insights into pathogenesis of early fatty liver disease in laying hens
Source: J Anim Sci Biotechnol. 2024 Mar 7;15:40. doi: 10.1186/s40104-024-01001-y (PMC10919017; doi:10.1186/s40104-024-01001-y)
Supplement: Supplementary file 2 — Additional file 2: Table S1. Forward and reverse primer sequences for RT-PCR analysis. [file 40104_2024_1001_MOESM2_ESM.docx]

**Table S1** Forward and reverse primer sequences for RT-PCR analysis

| Gene | Accession number | Primer sequences, 5′ to 3′ | Product size, bp |
| --- | --- | --- | --- |
| *β-actin* | L08165 | F: ATTGTCCACCGCAAATGCTTC | 113 |
|  |  | R: AAATAAAGCCATGCCAATCTCGTC |  |
| *MTHFR* | XM417645.3 | F: CGAACCCATCAAGGATAACGA | 141 |
|  |  | R: TCAGTAGTAGCCACTTCCCGATT |  |
| *MTR* | XM040663386.2 | F: CCTCCCATCTCTCAGTCTGC | 106 |
|  |  | R: TCAGTGAGGACAGGTGAGGA |  |
| *FAS* | NM_205155 | F: TTTGGTGGTTCGAGGTGGTA | 212 |
|  |  | R: CAAAGGTTGTATTTCGGGAGC |  |
| *ACACA* | XM_046929960 | F: GCTTCCCATTTGCCGTCCTA | 185 |
|  |  | R: GCCATTCTCACCACCTGATTACTG |  |
| *ELOVL6* | XM_046916529 | F: GGTGGTCGGCACCTAATGAA | 169 |
|  |  | R: TCTGGTCACACACTGACTGC |  |
| *DNMT1* | NM206952.1 | F: ACGGCTTCTTCAGCACCACGGTC | 168 |
|  |  | R: CTGTCGGTGTTTATCCAGGATGTT |  |
| *CBS* | XM_040659743.2 | F: GTGTCTAAGGCCAAGCCAGA | 229 |
|  |  | R: TGGGCAGGATATTTGGAGGC |  |
| *CPT1A* | XM_046918285 | F: TAGAGGGCGTGGACCAATAA | 229 |
|  |  | R: TGGGATGCGGGAGGTATT |  |
