# Supplementary material for: Reorganization of 3D genome architecture provides insights into pathogenesis of early fatty liver disease in laying hens
Source: J Anim Sci Biotechnol. 2024 Mar 7;15:40. doi: 10.1186/s40104-024-01001-y (PMC10919017; doi:10.1186/s40104-024-01001-y)
Supplement: Supplementary file 3 — Additional file 3: Fig. S1. KEGG pathways analysis based targeted genes from gain (A) and loss (B) H3K27ac peaks as well as differential peaks in promoter regions (C for gain and D for loss). Fig. S2. Gene expression from RT-PCR and DNA motif prediction. A–D Hepatic ACACA, FASN, ELOVL6 and CPT1A expression respectively. E–G DNA motif analysis for differential chromatin loops, loss and gain H3K27ac peaks in the promoter regions respectively. H–K Hepatic MTHFR, MTR, DNMT1 and CBS expression. All data for gene expression are showed as mean ± SEM (n = 7). The asterisks on the bars are statistically significant (* means P < 0.05 and ** means P < 0.01). [file 40104_2024_1001_MOESM3_ESM.docx]

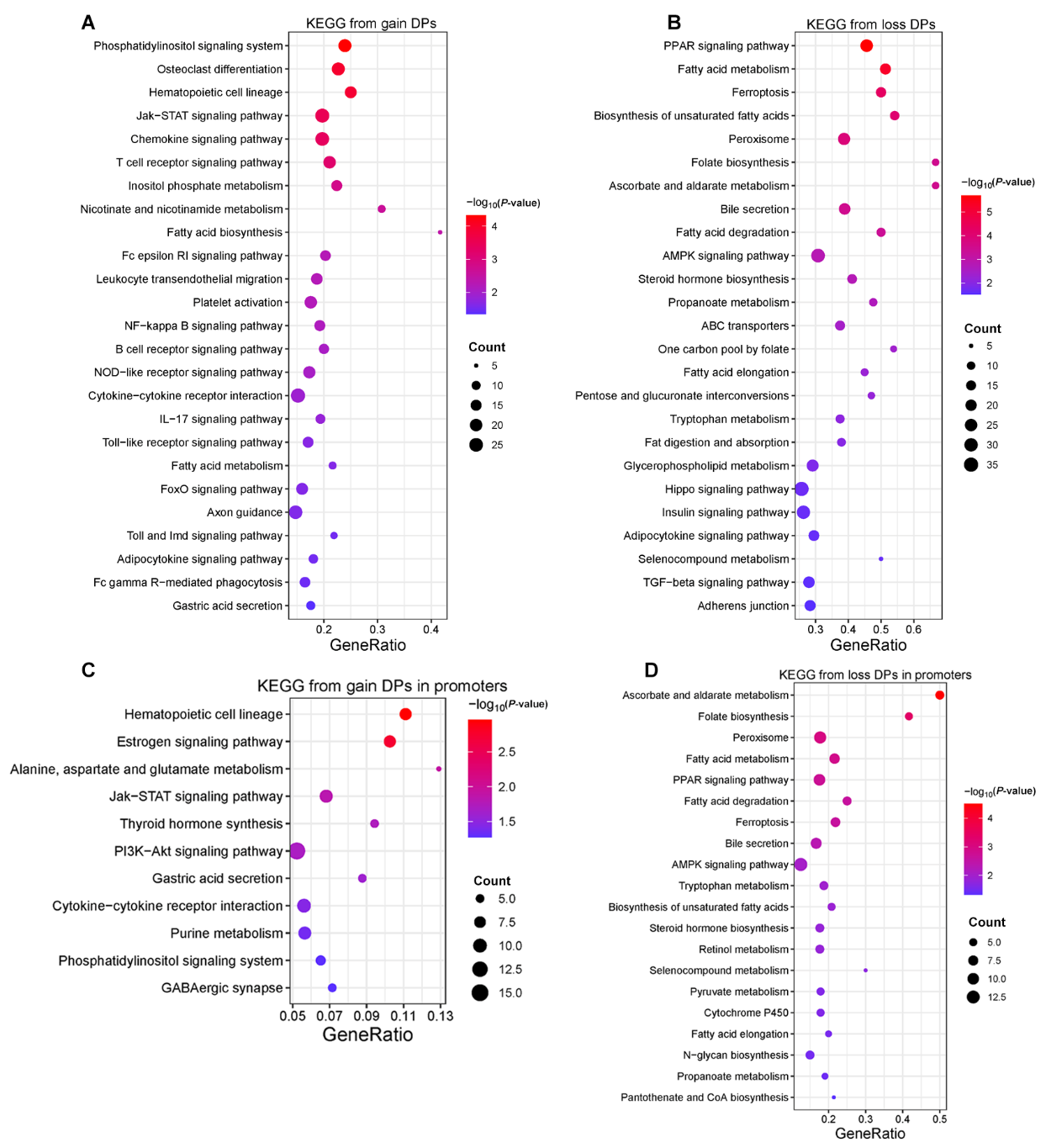


**Fig. S1** KEGG pathways analysis based targeted genes from gain (**A**) and loss (**B**) H3K27ac peaks as well as differential peaks in promoter regions (**C** for gain and **D** for loss)


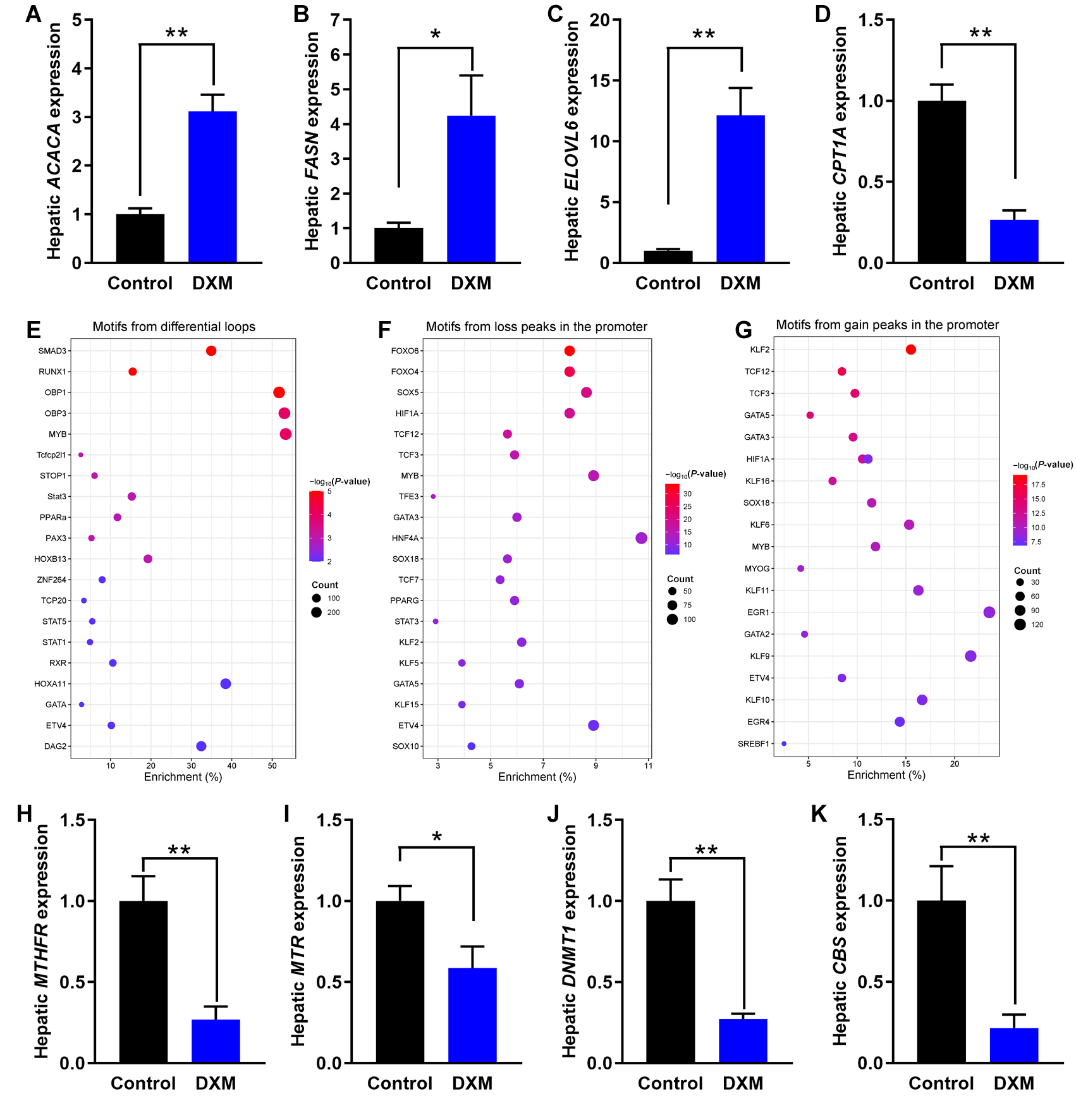


**Fig. S2** Genes expression from RT-PCR and DNA motif prediction. **A**–**D** Hepatic *ACACA*, *FASN*, *ELOVL6* and *CPT1A* expression respectively. **E**–**G** DNA motif analysis for differential chromatin loops, loss and gain H3K27ac peaks in the promoter regions respectively. **H**–**K** Hepatic *MTHFR*, *MTR*, *DNMT1* and *CBS* expression. All data for gene expression are showed as mean ± SEM (*n* = 7). The asterisks on the bars are statistically significant (* means *P* < 0.05 and ** means *P* < 0.01)
